# Effective contact tracing for COVID-19: A systematic review

**DOI:** 10.1016/j.gloepi.2023.100103

**Published:** 2023-03-09

**Authors:** Carl-Etienne Juneau, Anne-Sara Briand, Pablo Collazzo, Uwe Siebert, Tomas Pueyo

**Affiliations:** a Direction régionale de santé publique, CIUSSS du Centre-Sud-de-l'Île-de-Montréal, Montréal, Québec, Canada; b École de santé publique, Université de Montréal, Montréal, Québec, Canada; c Danube University Krems, Dr. Karl Dorrek-Strasse 30, 3500 Krems, Austria and IEEM Universidad de Montevideo, Lord Ponsonby 2542, 16000 Montevideo, Uruguay; d Institute for Technology Assessment, Department of Radiology, Massachusetts General Hospital, Harvard Medical School, Boston, MA, USA; e Department of Public Health, Health Services Research and Health Technology Assessment, UMIT - University for Health Sciences, Medical Informatics and Technology, Austria; f COVID-19 Work Group, USA

**Keywords:** COVID-19, Systematic review, Contact tracing, Effectiveness, Outbreak control, Epidemic control

## Abstract

Contact tracing is commonly recommended to control outbreaks of COVID-19, but its effectiveness is unclear. Following PRISMA guidelines, we searched four databases using a range of terms related to contact tracing effectiveness for COVID-19. We found 343 papers; 32 were included. All were observational or modelling studies. Observational studies (*n* = 14) provided consistent, very-low certainty evidence that contact tracing (alone or in combination with other interventions) was associated with better control of COVID-19 (e.g. in Hong Kong, only 1084 cases and four deaths were recorded in the first 4.5 months of the pandemic). Modelling studies (*n* = 18) provided consistent, high-certainty evidence that under assumptions of prompt and thorough tracing with effective quarantines, contact tracing could stop the spread of COVID-19 (e.g. by reducing the reproduction number from 2.2 to 0.57). A cautious interpretation indicates that to stop the spread of COVID-19, public health practitioners have 2–3 days from the time a new case develops symptoms to isolate the case and quarantine at least 80% of its contacts.

## Introduction

Late in 2019, the first outbreak of coronavirus disease (COVID-19) was discovered in Wuhan, China. On March 11, 2020, the World Health Organization (WHO) characterised COVID-19 as a pandemic. In April and May 2020, it published guidelines on public health control measures, including contact tracing [1].

Contact tracing involves identifying, notifying, and quarantining people who have had close contact with new cases in order to prevent further transmission within the community [1]. Prior to COVID-19, it had been used for other infectious diseases, such as HIV, tuberculosis, Ebola, SARS, and H1N1 influenza, for which it was estimated 4363 times more cost-effective than school closure ($2260 vs. $9,860,000 per death prevented) [2]. In May 2020, initial WHO guidelines for contact tracing were: “At least 80% of new cases have their close contacts traced and in quarantine within 72 hours of case confirmation.” [1] However, some studies already suggested that tracing needed to be more thorough and prompt to be effective [3,4]. WHO guidelines did not cite peer-reviewed evidence.

The US and European Centres for Disease Control and Prevention (CDC) also recommended contact tracing, but offered seemingly conflicting advice in the face of widespread transmission, when thousands of daily new contacts must be traced. Indeed, in May 2020, the US CDC wrote: “When a jurisdiction does not have the capacity to investigate a majority of its new COVID-19 cases, [it] should consider suspending or scaling down contact tracing.” [5] In contrast, in April 2020, the European CDC advised: “Contact tracing should still be considered in areas of more widespread transmission, wherever possible, and in conjunction with physical distancing measures.” [6,7] This apparent contradiction and lack of evidence in official recommendations may have left contact tracers wondering which guidelines to follow, and how effective tracing was. Case in point: the UK spent ten billion pounds on its test and trace programme, which may not have been effective due to limited case detection and compliance in naming contacts [8]. Therefore, in this systematic review, we aimed to examine contact tracing effectiveness and to identify characteristics of effective tracing efforts.

## Methods

We prepared this systematic review in accordance with PRISMA guidelines [9]. Its protocol was registered with PROSPERO (CRD42020198462).

### Eligibility criteria

All studies evaluating the effectiveness of contact tracing efforts in the community (alone or in combination with other interventions) were included. Effectiveness was defined as stopping or slowing the spread of COVID-19, or reducing the burden of infection. As Nussbaumer-Streit et al. [10] have, we operationalised “burden of infection” as including symptoms, complications, disability, hospitalisation, health-related quality of life, unintended health-related harms of interventions, infected individuals, and reproduction numbers. Randomised trials, observational studies, and modelling studies were included. All methods of contact investigation were considered, including tracing by telephone or via mobile apps. Articles in English, French, Spanish, and Portuguese from all countries were included. Studies were included only if they focused on SARS-CoV-2, as this virus poses unique challenges (such as asymptomatic and presymptomatic transmission). Abstracts, letters, protocols, preprints, and other unreviewed research were excluded, as well as studies limited to hospitals, nursing homes, prisons, and other enclosed spaces where transmission dynamics may not reflect that of the community. Reviews were also excluded, but their reference lists were checked for additional studies.

### Search strategy

MEDLINE (1946–2020 July 8), Embase (1974–2020 July 10), Global Health (1973–2020 Week 26), and All EBM Reviews (2005–2020 July 10) were searched using the terms “COVID-19” OR “coronavirus disease 2019” OR “SARS-CoV-2” OR “severe acute respiratory syndrome coronavirus 2” OR “2019-NCoV” OR “2019 novel coronavirus” AND “contact tracing” OR “contact-tracing” OR “tracing contact*” OR “contact follow-up” OR “case detection*” OR “contact investigation*” OR “epidemic investigation*” with no language or date restrictions. To find additional articles, we also reviewed reference lists, used the “related articles” and “cited by” functions in Google Scholar and PubMed, searched our own files, and consulted with colleagues.

### Data extraction and synthesis

Two investigators screened all studies. Discrepancies were solved by mutual agreement. Characteristics of studies were recorded in a spreadsheet. Those included: first author, publication date, study design, population, characteristics of contact tracing efforts, and main findings. We found substantial differences in study design, settings, outcomes, and effect measures. For example, in some settings, contact tracing was implemented along with lockdowns and other interventions (e.g., China). In other settings, it was not (e.g., South Korea). In addition, across studies, effect measures varied widely. We concluded that meta-analysis was not feasible [11]. Therefore, to promote transparent reporting, we followed synthesis without meta-analysis (SWiM) guidelines [12]. We synthesised results using the vote-counting method and narratively. For narrative synthesis, we focused on studies with the lowest risk of bias. We reported results separately for observational and modelling studies in a GRADE evidence profile table [13].

### Risk of bias

We assessed risk of bias using methods similar to a Cochrane review on a related topic (quarantines for COVID-19) [13]. Briefly, for observational studies, a Cochrane tool was used [14]. This tool assesses internal and external validity across eight criteria (Table 1). Overall risk of bias was then rated as low (when all validity criteria were met), moderate (when at least one criterion was unclear), or serious (when at least one criterion was not met). For modelling studies, we used three criteria for best practices recommended by the International Society for Pharmacoeconomics and Outcomes Research (ISPOR) and the Society for Medical Decision Making (SMDM). These were: (1) Was the model dynamic? A model is dynamic when infection risk at a given point in time is dependent on the number of infectious individuals [15]. (2) Were uncertainty analyses conducted on key model parameters and assumptions? Uncertainty analyses quantify the influence of variations in model inputs on predicted effects (3) Did results include a comparison of the burden of infection? Overall, we had no concerns to minor concerns when a modelling study followed all three criteria. If at least one practice was unclear, we had moderate concerns. Lastly, if at least one practice was not followed, we had major concerns. Two investigators independently assessed risk of bias. Discrepancies were solved by mutual agreement.

**Table 1:** Risk of bias criteria for single-arm observational studies of interventions.

	Internal validity	External validity
Study group	Selection bias (representative: yes/no)	Reporting bias (well defined: yes/no)
	Yes if the described study group consisted of >90% of eligible individuals	Yes if the intervention and number of participants was defined
Follow-up	Attrition bias (adequate: yes/no)	Reporting bias (well defined: yes/no)
	Yes if the outcome was assessed for at least 60% of the study group of interest	Yes if the length of follow-up was mentioned
Outcome	Detection bias (blind: yes/no)	Reporting bias (well-defined: yes/no)
	Yes if the outcome assessors were blinded to the investigated determinant	Yes if the outcome definition was objective and precise
Risk estimation	Confounding (adjustment for other factors: yes/no)	Analyses (well-defined: yes/no)
	Yes if important prognostic factors (i.e. age, gender) or follow-up were taken adequately into account	Yes if the method of analysis was described and the effect of the intervention was quantified

### Certainty of evidence

We rated the certainty of evidence using GRADE [13]. This approach was also used by a Cochrane review on a related topic (quarantines for COVID-19) [10]. Briefly, for observational studies, the evidence starts as low-certainty. For modelling studies, it starts as high-certainty. It can then be adjusted according to risk of bias, indirectness, inconsistency, imprecision, and publication bias. Overall, the evidence can be graded as high-certainty (very confident that the true effect lies close to the estimated effect), moderate-certainty (moderately confident in the effect estimate), low-certainty (limited confidence: true effect may be substantially different), or very low-certainty (very little confidence: true effect likely substantially different). One investigator rated the certainty of evidence. A second investigator checked the ratings.

## Results

### Result of the search

A total of 544 papers were found. Removing duplicates left 343. We retained 158 based on title, 64 based on abstract, and 27 based on full text. We found one additional study via reference lists and four more via the “cited by” and “similar articles” functions of PubMed and Google Scholar (eFigure in the Supplement). Therefore, this systematic review includes 32 studies (Table 2) [3,4,[16], [17], [18], [19], [20], [21], [22], [23], [24], [25], [26], [27], [28], [29], [30], [31], [32], [33], [34], [35], [36], [37], [38], [39], [40], [41], [42], [43], [44], [45], [46], [47], [48], [49], [50], [51], [52], [53], [54], [55], [56]].

**Table 2:** Characteristics of the 32 studies included in this systematic review.

Last name of first author	Publication date	Study design	Setting and population	Characteristics of tracing efforts	Key finding(s)
Burke	03–2020	Observationnal study	United States	Identification of close contacts and 14 days active symptom monitoring	- Contact tracing in the US started on January 20th - The first 10 travel-related COVID-19 cases had 445 close contacts. Among those contacts, two developed COVID-19 and had 146 additionnal contacts. - There was no sustained person-to-person transmission among the close contacts of the first 10 persons with diagnosed travel-related COVID-19.
Choi	05–2020	Observationnal study	South Korea	Combination of contact tracing, quarantine, testing, isolation, social distancing and school closure.	- As of 23 March 2020, extensive testing, contact tracing, quarantine and isolation led to effective control of the outbreak in the main affected areas of South Korea (Daegu and Gyeongsangbuk).
Lam	06–2020	Observationnal study	Hong Kong	Combination of border control, social distancing, enhanced surveillance and contact tracing	- Border control, social distancing, enhanced surveillance and contact tracing resulted in the successful prevention of community-wide outbreak and containment of the epidemic in Hong Kong. - Contact tracing allowed to quickly contain several local clusters associated with bars and social gatherings and resulted in transient and limited community spread.
Choi	06–2020	Observationnal study	South Korea	Extensive digital surveillance technologies	- South Korea's public health authorities have used extensive digital surveillance technologies to support their contact tracing, such as tracked mobile phone location data, analysis of security camera footage (in private and public spaces) and tracking of credit card statements. - The citizen's tolerance to privacy infringement is one unique condition that must have contributed to the success of South Korea's measures, in addition to the MERS experience from 2015.
Nachega	05–2020	Observationnal study	Rwanda, South Africa, Tanzania, the Democratic Republic of Congo and Mozambique	Depending on the country: tracing by telephone, SMS, and/or home visits, and quarantine or monitoring of contacts, sometimes in combination with travel bans and lockdowns	- Rwanda, South Africa, Tanzania, the Democratic Republic of Congo and Mozambique have implemented contact tracing as part of their response to COVID-19. - Multiple strategies have been used to trace contacts, including provincially based telephonic teams (South Africa), short messaging systems and home visits (Tanzania). - In Rwanda, contacts (<1 m) are traced for a period 2 to 14 days before the onset of the symptoms of the case. - Rwanda and Mozambique interventions have proven effective so far.
Bernard Stoecklin	02–2020	Observationnal study	France	As of February 2020 in France, initial questionning of cases for contact tracing was done by clinicians in collaboration with regional health entities and Santé Publique France. Contacts were stratified in different risk categories and measures were taken accordingly. Active surveillance and home quarantine was applied to moderate-high risk contacts.	- Most contacts at risk were identified for the first 3 cases, but some low risk contacts could not be traced (e.g co-travellers on public transportation). No secondary transmission was detected. - Contact tracing and follow-up was rapid and collaborative.
Chen	05–2020	Observationnal study	Taiwan	Contact tracing using data from GPS in the shuttle bus, credit card transaction log, closed-circuit television (CCTV), and mobile position data	- Tracing of taiwanese citizens possibly exposed to passengers on the Diamon Princess was done by using travelling itinerary arranged by the agency, GPS in shuttle buses, credit card transaction logs, closed circuit televisions, vehicle license plate recognition system, and mobile positioning data. - 627,386 citizens were sent syndrome monitoring and self-quarantine information via SMS messaging. - National Health Insurance Claims data was used to track the health status of all contacts. No COVID positive case was identfied.
Ng	03–2020	Observationnal study	Singapore	Combination of case detection and isolation, contact tracing, border controls, and community education	- Multiple strategies were adopted by Singapore to control the outbreak since January (case detection and isolation, contact tracing, border controls, and community education) - Among the first 100 COVID-19 patients, 53 were identified through contact tracing, of which 13 (24.5%) were contacted on or before the date of symptom onset. - Low risk contact were followed once daily and close contacts thrice daily, for symptom monitoring. - The interval from symptom onset to isolation declined significantly across the study period (January–February 2020). - The number of newly identified cases decreased after approximately 1 month, likely as a result of the early implementation of surveillance and detection measures.
Wong	05–2020	Observationnal study	Hong Kong	Mandatory quarantine of close contacts in quarantine centre or at home with electronic wristbands to assess compliance	- Despite being particularly vulnerable to COVID-19 (high population density, regional travel hub, proximity to China), Hong Kong has been able to control the disease spread. - Public health measures, including contact tracing, quarantine centres, border control, social distancing and high-volume testing, likely contributed substantially to the control of the outbreak, especially during the early period. - Quarantine of close contacts was mandatory. Several quarantine centres were open. Some close contacts self-quarantined with monitoring of compliance by electronic wristbands.
Dinh	04–2020	Observationnal study	Vietnam	Tracing of contacts up to 5 generations	- Vietnam has kept its outbreak under control for 2 months using different measures, including case finding, contact tracing, border controls, social distancing and population use of masks. - In terms of contact tracing, once a case was identified, contacts were traced up to five generations.
Bi	04–2020	Retrospective cohort study	Shenzhen, China	- Close contacts were those who lived in the same apartment, shared a meal, travelled, or socially interacted with an index case 2 days before symptom onset. Casual contacts (eg, other clinic patients) and some close contacts (eg, nurses) who wore a mask during exposure were not included. - All close contacts were tested at the beginning and end of isolation.	- Contact-based surveillance was associated with a 2·3-day decrease in time to confirmation and a 1·9-day decrease in time to isolation. - The reduction of time during which cases are infectious in the community should reduce the R. However, the overall impact is highly dependent on the number of asymptomatic cases.
Cowling	05–2020	Observational study	Hong Kong	Combination of testing and isolating cases, tracing and quarantining contacts, and some social distancing	- The experience in Hong Kong indicates that COVID-19 transmission can be contained with a combination of testing and isolating cases, plus tracing and quarantining their close contacts, along with some degree of social distancing to reduce community transmission from unidentified cases.
Wilasang	06–2020	Observationnal study	Cross-country analysis	testing with active case finding and prompt isolation, combined with contact tracing and quarantine	- Among 10 selected countries (Belgium, China, France, Germany, Iran, South Korea, Spain, Thailand, USA and UK), only China and South Korea achieved a > 2 decrease in Rt in <3 weeks. - China's approach (strict lockdown with case-finding and physical distancing) or South Korea's approach (no lockdown, but rigorous prompt case-finding and quarantine of all contacts) may be key to successful outbreak control. - Other hallmark measures taken in China and South Korea: house-to-house surveys, drive-in testing, policies for isolation of all cases, enhanced contact tracing with digital technologies and close monitoring of all quarantined contacts to identify new cases.
Davalgi	04–2020	Observationnal study	Cross-country analysis (Germany, Italy, USA, Canada, China, Singapore, Iran, Saudi Arabia, India, Sri Lanka)	Extensive testing and contact tracing	- Countries with extensive testing and contact tracing like China, Singapore, South Korea and Germany showed better outcomes than countries with limited testing like Italy.
Zu	06–2020	Modelling study	China, based on data sets from January 23, 2020 to February 17, 2020	Quarantine rate of close contacts	- Important decrease of cases and deaths with increased contact-tracing and quarantine. The quarantined rate was estimated to be 26.53% in the first month of the pandemic in China. With a rate of 50%, there would have been a decrease of 62% of cumulative cases and deaths (88% with a 100% quarantined rate). - Reproduction number went from 2.620 on January 23rd to below 1.0 since February 5th. - Without measures, there would have been 38,364,910 additionnal cases at the peak around March 11th.
Tang	05–2020	Modelling study	Ontario (Canada)	Quarantine rate of close contacts	- 1st-2nd de-escalation phase: Rc < 1 only if 60% of contacts are quarantined (q = 0.6), but could be less if some social distancing is maintained. - Improving diagnosis rate has limited effect on reduction of Rc, unless combined with improved isolation and quarantine. - 3rd de-escalation phase: assuming the same diagnosis and contact rate, only unrealistically high values of the quarantine rate would avoid a rebound. With a quarantined rate of 50%, a reduction in contact of 50% would keep Rc < 1.
Wu	05–2020	Modelling study	Ontario (Canada), based on data sets from February 26, 2020 to March 29, 2020	Quarantine rate of close contacts	- Decreasing number of cumulative cases and peak with increased quarantine rate - Rc can be reduced rapidly under 1 with a combination of increased diagnosis and quarantine rate. - Contact rate, transmission probability, detection/diagnose rate, and quarantine rate are key factors that influence the control reproduction number
Maier	05–2020	Modelling study	China	Analysis of the cumulative cases observed in relationship to the implementation of different measures by the government, such as lockdown, social distancing, case isolation and contact tracing.	- Containment measures put in place by the chinese government during the first wave of COVID-19, including social distancing and contact tracing, were effective in containing the outbreak.
Ngonghala	05–2020	Modelling study	United States	Population level impact of four main intervention strategies (social-distancing, quarantine/isolation, contact-tracing and the use of face-masks)	- Only a small decrease in the burden of the pandemic was seen with the highest possible level of contact-tracing at pandemic peak (13% and 10% for cases in New York state and nationwide, respectively, and 5% and 3% for mortality for New York state and nationwide, respectively). - Contact-tracing is marginally-effective in minimizing the burden of the pandemic and might not be cost-effective.
Giordano	04–2020	Modelling study	Italy	Combination of population-wide testing and contact tracing in addition to lockdown	- With increased population-wide testing and contact tracing efforts, the peak of the pandemic could be reached sooner and the number of deaths lessened.
Kucharski	06–2020	Modelling study	United Kingdom	Analysis of the effective reproduction number in relationship to the implementation of different measures by the government, such as self-isolation of symptomatic cases; household quarantine; manual tracing of acquaintances (ie, contacts that have been met before); manual tracing of all contacts; app-based tracing; mass testing regardless of symptoms; limits on daily contacts made outside home, school, and work; and having a proportion of the adult population work from home	- The Reff is lower with combined isolation of symptomatic cases and contact tracing (0.94) than with mass testing (2.5) or self-isolation alone (1.8). In comparison to a Reff of 2.6 with no measures. - The effectiveness of this combination can be increased with the addition of physical distancing measures and app-based tracing (0.87). - Contact tracing also substantially reduce the probability that a primary case generate many secondary cases. - Effectiveness of manual contact-tracing strategies is highly dependent on the number of successfully traced contacts. High level of tracing required to ensure Reff <1. - App-based tracing would require a high level of coverage to ensure Reff <1.
Gosce	06–2020	Modelling study	London, United Kingdom	Lockdown, weekly universal testing, case isolation, contact tracing and facemask use	- Compared to lockdown alone, adding weekly universal testing, case isolation, contact tracing and facemask use is much more effective in controlling the outbreak and reducing lockdown length. - This combination could reduce cumulative deaths by 48% compared with lockdown alone.
Tang	02–2020	Modelling study	China	Quarantine rate of contacts	- Increasing quarantine rate by 10 or 20 times could bring forward the peak by 6.5 or 9 days, and lead to a reduction of the peak number of infected individuals by 87% or 93%. - The number of contacts traced in Wuhan as of 22 January 2020 (estimated to be 5897) was insufficient compared to the population size and appears to have had a limited impact on the epidemic control. - The quarantine rate needs to be very high for a city to avoid an outbreak. - The duration of travel restriction depends on a combination of effective quarantine and reduction of contact within the city.
Lai	05–2020	Modelling study	China	Travel restrictions, early identification and isolation of cases (including contact tracing), and social distancing	- Early detection and isolation of cases was estimated to quickly and substantially prevent more infections than contact reduction and social distancing across the country (5-fold versus 2.6-fold). - Combined interventions achieved the strongest and most rapid efect.
Tang	03–2020	Modelling study	Wuhan	Lock-down, contact tracing followed by quarantine and isolation	- Under the strict prevention and control measures, the effective daily reproduction ratio has been <1 since January 26th, 2020 - Our updated findings suggest that the best measure is persistent and strict self-isolation.
Currie	06–2020	Modelling study	Australia	COVIDSafe app-based contact tracing with varying levels of population uptake (0%, 27%, 40%, 61%, 80%), social distancing (50% decline every 3 weeks, month, or 6 weeks), and testing intensity (no decline, 5% decline, and 10% decline every month)	- The low Australian COVID-19 incidence and mortality during February–May 2020 are probably attributable to the multiple strategies used, including contact tracing. - The effect of the app increases as its uptake increases, to a disproportionately greater extent than the increment in uptake. - Some scenarios show that an app uptake of 61% has the potential to reduce the cumulative total number of new cases by >50%. - COVIDSafe would have the capacity to contribute susbtantially to contact tracing in a second wave and serve as an adjunct to testing and social distancing.
Ferretti	05–2020	Modelling study	Hypothetical population	Epidemic control with varying success in isolating cases (0–100%), in quarantining contacts (0–100%), and delays (0–3 days) between initiation of symptoms to case isolation and quarantine of contacts.	- The delay to isolation and contact quarantine is key to containing the pandemic - No delays to isolation and contact quarantine is associated with the greatest epidemic decline, whereas a 3 days delay would be associated with no decline. - A mobile phone app implementing instantaneous contact tracing could reduce transmission enough to achieve *R* < 1 and sustained epidemic suppression.
Hellewell	02–2020	Modelling study	Hypothetical population	Outbreak control with case isolation and varying number of initial cases (5, 20, 40), R0 (1.5, 2.5, 3.5), delay (3.43 or 8.09 days), and contacts traced (0%, 20%, 40%, 60%, 80%, 100%)	- The probability of an outbreak control with contact tracing is higher with a R0 of 1.5, and fall rapidly with a R0 of 3.5. It also drops as the number of initial cases are increased. - A high number of cases and contacts can overwhelm the contact-tracing system and affect the quality of the contact-tracing effort. - In most plausible outbreaks scenarios, case isolation and contact tracing alone is insufficient to control outbreaks within 3 months, but can contribute to reduce the overall size of the outbreak or to bring it under control over a longer time period.
Keeling	06–2020	Modelling study	United Kingdom	Hypothetical programme based on a survey of social encounters of 5802 respondents	- R0 decreased from 3 to 0.18 when 100% contacts traced. - R0 decreased from 3 to below 1 when 71% contacts traced. - Assumptions: infectious period = 2 days; all contacts traced before they infect other people (no tertiary cases)
Yasaka	04–2020	Modelling study	Hypothetical population	App-based contact tracing with varying levels of population adoption (0%, 25%, 50%, 75%)	- Development of a peer-to-peer smartphone app not using location data - Computer simulation model shows that adoption rate of the app determines its impact on the outbreak and that, if participation is low, the intervention will have limited effect.
Peak	05–2020	Modelling study	Hypothetical population	High-feasibility setting (90% contacts traced, 0.5 days delay, contacts monitored every 0.5 days, 75% reduction of infectiousness for contacts under quarantine, 90% reduction of infectiousness for cases under isolation) Low-feasability setting (50% contacts traced, 2 days delay, contacts monitored every 2 days, 25% reduction of infectiousness for contacts under quarantine, 50% reduction of infectiousness for cases under isolation)	- In a high-feasibility setting, the median effective reproductive number was 0·57 under individual quarantine with the shorter serial interval (4.8 days) and 0.49 with the longer serial interval (7.5 days) - In a low-feasibility setting, R under individual quarantine remained above 1 for both serial interval scenarios (4.8 and 7.5 days), even when R0 was 1·5 - Even with other operational parameters reflecting a high-feasibility setting, at least 75% of contacts need to be traced and quarantined to reduce R below 1, assuming a mean serial interval of 4.8 days and a basic R0 of 2.2. - Depending on the ratio of uninfected to infected contacts traced, individual quarantine may become infeasible as the epidemic grows, even if initially effective.
Kretzschmar	07–2020	Modelling study	Hypothetical population	Outbreak control with varying testing delays, tracing delays, contacts traced (0–100% or 80% of close contacts and 50% of casual contacts), and 40% of transmissions taking place before symptom onset	- With a testing delay of 3 days or longer, even the most efficient tracing could not bring the reproduction number below 1 - With testing and tracing delays of 0, 100% of contacts were traced, 40% of transmissions occurring before symptom onset, and no further transmission upon quarantine, reproduction number decreased from 1.2 to 0.8 (95% CI 0·7–0·9).

### Observational studies

Results of observational studies were consistent: 14 out of 14 (100%) reported that contact tracing (alone or in combination with other interventions) was associated with better control of COVID-19 [[16], [17], [18], [19], [20], [21], [22], [23], [24], [25], [26], [27], [28], [29]]. Outcomes measures included changes in daily new cases at the country or regional levels, reproduction numbers, doubling times, and serial intervals. Some studies had no measures of association at all (only descriptive statistics). We found serious risk of bias in 14 out of 14 observational studies (Table 3). Detection bias was present in all studies (14/14 studies). Potential bias due to confounding was the second most common (11/14 studies). In contrast, there was little selection bias (1/14 studies) and outcome reporting bias (0/14 studies). Overall, the certainty of this body of evidence was rated as very low (Table 4). Studies were carried out in China [26], Hong Kong [18,24,27], Taiwan [22], Singapore [23], South Korea [17,19] Vietnam [25], France [21], the U.S. [16] in African countries [20], and in international comparisons [28,29]. The six studies with the lowest risk of bias were carried out in China [26], Hong Kong [18,27], Taiwan [22], Singapore [23], and South Korea [17]. In all six studies, contact tracing was implemented early, along with border control measures and enhanced surveillance. In Hong Kong and South Korea, additional social distancing measures were also implemented. In Shenzhen, China, 391 cases and 1286 close contacts were identified over a 1-month period (January 14–February 12, 2020). Cases were isolated on average 4·6 days (95% CI 4.1–5.0) after developing symptoms; contact tracing reduced this delay by 1·9 days (95% CI 1.1–2.7) [26]. In Hong Kong, the first case was recorded on January 23, 2020. By May 31 (∼4.5 months later), the outbreak was controlled (no community transmission). Overall, only 1084 cases and four deaths were recorded in Hong Kong in the first 4.5 months of the pandemic—in a population of 7.4 million with high density and intimate ties with China [18]. In Taiwan, big data and mobile geopositioning were used to help trace 627,386 persons in contact with Diamond Princess cruise ship passengers. Of those, 67 contacts tested negative and no confirmed COVID-19 cases were found, indicating successful containment. In addition, during follow-up, respiratory syndrome and pneumonia were less common among traced contacts than in the general population [22].

**Table 3:** Risk of bias assessment of observational studies (n = 14).

Study first author	Year	Selection bias (representative study group: yes/no)	Attrition bias (complete follow-up assessment: yes/no)	Detection bias (blinded outcome assessor: yes/no)	Confounding (adjustment for important confounders: yes/no)	Reporting bias (well defined study group: yes/no)	Reporting bias (well defined follow-up: yes/no)	Reporting bias (well defined outcome: yes/no)	Analyses (well defined: yes/no)	Overall risk of bias
Bernard Stoecklin	2020	Yes	Yes	No	No	Yes	Yes	Yes	Yes	Serious
Bi	2020	Yes	No	No	Yes	Yes	Yes	Yes	Yes	Serious
Burke	2020	Yes	Yes	No	No	Yes	Yes	Yes	Yes	Serious
Chen	2020	Yes	Yes	No	Yes	Yes	Yes	Yes	Yes	Serious
Choi	2020	Yes	Yes	No	No	Yes	Unclear	Yes	Unclear	Serious
Choi	2020	Yes	Yes	No	Yes	Yes	Yes	Yes	Yes	Serious
Cowling	2020	Yes	Yes	No	Yes	Yes	Yes	Yes	Yes	Serious
Davalgi	2020	Yes	Yes	No	No	Yes	Unclear	Yes	Yes	Serious
Dinh	2020	Yes	Yes	No	No	Yes	Yes	Yes	Yes	Serious
Lam	2020	Yes	Yes	No	Yes	Yes	Yes	Yes	Yes	Serious
Nachega	2020	Yes	Yes	No	No	Yes	Yes	Yes	Yes	Serious
Ng	2020	Yes	Yes	No	Yes	Yes	Yes	Yes	Yes	Serious
Wilasang	2020	Yes	Yes	No	No	Yes	Yes	Yes	Yes	Serious
Wong	2020	Yes	Yes	No	No	Yes	Yes	Yes	Yes	Serious

**Table 4:** GRADE evidence profile.

Outcome	Number of studies	Risk of bias	Indirectness	Imprecision	Inconsistency	Other considerations	Summary of findings	Certainty of evidence
Control of COVID-19 (observational studies)	14	Serious	Direct	Precise	Consistent	None	Contact tracing was associated with better control of COVID-19 in 14/14 studies (100%)	Very low
Control of COVID-19 (modelling studies)	18	Low	Direct	Precise	Consistent	None	Under assumptions of prompt and thorough tracing with effective quarantines, contact tracing could stop the spread of COVID-19 in 18/18 studies (100%) Under assumptions of slower, less efficient tracing, contact tracing could slow, but not stop COVID-19	High

### Modelling studies

Results of modelling studies (*n* = 18) depended on their assumptions. Peak et al. [46] illustrated this clearly by examining two sets of assumptions (in a study with a low risk of bias). In the first set of assumptions, 90% of contacts were traced within 0.5 days, quarantine reduced infectiousness by 75%, and isolation of cases reduced infectiousness by 90%. In this “high-feasibility setting”, the reproduction number was reduced from 2.2 to a median of 0.49–0.57, and the epidemic was controlled. In a second, “low-feasibility setting”, 50% of contacts were traced in 2 days, quarantine reduced infectiousness by 25%, and isolation of cases reduced infectiousness by 50%. In that setting, the epidemic was not controlled in any scenario. We examined a subset of five similar studies that modelled steps of the contact tracing process [3,4,43,44,46]. In those, under optimistic assumptions, efficient contact tracing always led to control of COVID-19. However, when less optimistic assumptions were modelled, inefficient contact tracing could at best slow, but not stop, outbreaks of COVID-19. For efficient tracing, these assumptions included short delays of 2–3 days from the time a new case develops symptoms to isolation of the case and quarantine of its contacts [3,44], at least 80% of contacts traced [3,4,43,44], and no further transmission upon isolation and quarantine [3,4,44,46]. In terms of quality, we had major concerns for 2/18 modelling studies (11%), moderate concerns for 1/18 study (6%) and no to minor concerns for 15/18 studies (83%) (Table 5). Overall, the certainty of this body of evidence was rated as high (Table 4). This rating should be interpreted with caution, however, as results of modelling studies were highly dependent on their assumptions.

**Table 5:** Risk of bias assessment of modelling studies (n = 18).

Study first author	Year	Was the model a dynamic (transmission) model?	Did the authors conduct uncertainty analyses on key assumptions that may have had an impact of the conclusions?	Do the results provide estimates of the change in the burden of infection due to the intervention?	Quality
Peak	2020	Yes	Yes	Yes	No to minor concerns
Currie	2020	Yes	Yes	Yes	No to minor concerns
Ferretti	2020	Unclear	Yes	Yes	Moderate concerns
Giordano	2020	Yes	Yes	Yes	No to minor concerns
Goscé	2020	Yes	Yes	Yes	No to minor concerns
Hellewell	2020	Yes	Yes	Yes	No to minor concerns
Keeling	2020	Unclear	Yes	No	Major concerns
Kretzschmar	2020	Yes	Yes	Yes	No to minor concerns
Kucharski	2020	Yes	Yes	Yes	No to minor concerns
Lai	2020	Yes	Yes	Yes	No to minor concerns
Maier	2020	Yes	Yes	No	Major concerns
Ngonghala	2020	Yes	Yes	Yes	No to minor concerns
Tang	2020	Yes	Yes	Yes	No to minor concerns
Tang	2020	Yes	Yes	Yes	No to minor concerns
Tang	2020	Yes	Yes	Yes	No to minor concerns
Wu	2020	Yes	Yes	Yes	No to minor concerns
Yasaka	2020	Yes	Yes	Yes	No to minor concerns
Zu	2020	Yes	Yes	Yes	No to minor concerns

## Discussion

This systematic review aimed to examine contact tracing effectiveness and to identify characteristics of effective tracing programmes. Observational studies (*n* = 14) provided consistent, very-low certainty evidence that contact tracing (alone or in combination with other interventions) was associated with better control of COVID-19. Modelling studies (*n* = 18) provided consistent, high-certainty evidence that under assumptions of prompt and thorough tracing with effective quarantines, contact tracing could stop the spread of COVID-19. This conclusion was supported by a number of preprints and other unpublished work [[38], [39], [40], [41], [42], [43], [44], [45], [46], [47], [48], [49], [50]]. However, under assumptions of slower, less efficient tracing, modelling studies found that tracing could slow, but not stop COVID-19.

Our results are in line with those of other reviews. A recent Cochrane review of quarantine (alone or in combination with other public health measures) found that modelling studies consistently reported a benefit of quarantine to control COVID-19, and that early implementation of quarantine and combining quarantine with other public health measures were important to ensure effectiveness [10]. Likewise, a narrative review of contact tracing in patients infected with SARS-CoV-2 concluded that this “classic” strategy could be applied, but that it should be accelerated [51].

According to modelling studies, if an outbreak is left unchecked for >2–3 days, it becomes practically impossible to stop with contact tracing alone (at this stage, tracing alone may only slow its spread) [3,54]. Therefore, timing and speed are crucial early on in an outbreak, and it stands to reason that as part of pandemic preparedness, contact tracers should be trained ahead of time, and proper settings and monitoring should be put in place to react in a timely manner. Ideally, tracing of contacts should begin as soon as the first case is detected in a jurisdiction. New cases can transmit the virus to others while presymptomatic or asymptomatic, further highlighting the importance of speed [3,4,53,54,56]. Cases should be tested and their results communicated in the shortest possible time. If new cases are not tested, wait to be tested, or receive test results after much delay, contact tracing is less effective. Thus, testing should be widely available, and the public could be reminded to seek testing at the earliest signs of symptoms. Likewise, systemic delays in communicating test results should be minimised. In the US, one report of drive-through testing found that when outsourced, tests (*n* = 476) were turned around in a median of 9.21 days [42]. Contact tracing may not be effective with such delays [3,54].

Once a case is confirmed, its contacts must be traced. When tracing contacts manually over the telephone, cases may not disclose, remember, or have contact information for all contacts. For example, a pilot programme in Sheffield, UK, found that two thirds of people contacted through tracing did not fully cooperate [53]. Moreover, during a peak of COVID-19, thousands of new contacts may need to be traced daily. Large contact tracing efforts with new staff are costly, and may not be able to maintain the level of effectiveness of smaller programmes with experienced staff. Mobile phone apps and other technologies can circumvent these shortcomings, but raise a number of ethical issues [54,55] and pose technological challenges [56]. Moreover, early evidence suggests that contact tracing apps, despite wide encouragement, have limited adoption [57,58] Thus, they may slow (but not stop) COVID-19.

Cases should isolate, and contacts should quarantine. Four of the five modelling studies we have reviewed assumed perfect prevention of transmission at these steps [3,4,53,54] This may be difficult to achieve in practice, even if cases and contacts never leave the home. Indeed, Bi et al. [26] found that household secondary attack rate was 11.2% (95% CI 9.1–13.8) in Shenzhen, China. Similarly, Park et al. [59] found that 11.8% of household contacts had COVID-19 in South Korea. Moreover, Wu and McGoogan [35] report that in 20 Chinese provinces outside of Hubei, a total of 1183 case clusters were found, of which 64% were within familial households. Thus, it may be more effective to isolate and quarantine outside the home, in hotels or central locations, especially considering many homes (1 in 5 in the US) will lack sufficient space to comply with recommendations [60]. If home isolation and quarantine are used nonetheless, information could be provided to new cases and contacts to reduce household transmission. Li et al. [61] followed 105 index patients and 392 household contacts in Wuhan, China. They found 14 cases had isolated by themselves at home immediately after the onset of symptoms—with masks, dining separately, and residing alone. No households contacts were infected. A final consideration for this step of the process is financial and social support. Contacts may need support for lost wages, daily activities carried out outside the home (e.g. groceries), or questions related to their health (e.g. telephone helpline).

### Strengths and limitations

This study has a number of strengths. To our knowledge, it is the first to systematically review the effectiveness of contact tracing efforts. It included both observational and modelling studies, hence examining contact tracing in two complementary ways. Observational studies examined how contact tracing (alone or in combination with other interventions) was associated with the spread of COVID-19. Modelling studies examined how varying assumptions about each step of the tracing process might influence its effectiveness.

This study also has a number of limitations. It was not feasible to perform a meta-analysis, so no synthesised estimates of the magnitude of associations are available. Moreover, observational studies reported results of contact tracing efforts in combination with other interventions. So, its independent potential effect could only be examined in modelling studies. Another limitation is the rapidly evolving nature of the pandemic and related contact tracing policies and regulations, as well as public perceptions, which are all likely to influence future tracing efforts. A final limitation is we did not discuss implementation. Rajan et al. [62] describe specific challenges and solutions when implementing contact tracing programmes.

This body of literature also has a number of limitations. These may be inherent, as studying contact tracing in the midst of a pandemic poses substantial methodological challenges. Indeed, we found no randomised controlled trials. This is perhaps not surprising, as lack of feasibility and ethical considerations arguably precludes them. We could not therefore draw conclusions about causality. The strength of our conclusions was further limited by the quality of the observational studies we found. All had a single arm, and none mentioned blinding of assessors. This increased risk of bias, according to the Cochrane tool we used. While we aimed to enhance replicability and standardization by using this tool, one may wonder if multiple arms and blinding of assessors are truly feasible during a pandemic. If not, specific tools may be required to assess the quality of pandemic studies, taking into account the exceptional circumstances in which they are carried out. Another limitation was that most observational studies (11 out of 14) did not account for potential confounders. What were the roles of age and gender? Of varying levels of compliance in naming contacts? Of other concurrent public health interventions, like masking and lockdowns? The complex interplay of these potential confounders and covariates limit our understanding of the true underlying dynamics of transmission from person to person and from group to group, which may change across settings, times, and concurrent interventions (e.g., from children in school to their home and play environment; from residents in nursing facilities to staff and home environments that are geographically dispersed, etc.). For all these reasons, while results of modelling studies may be encouraging, the connection between contact tracing and epidemic trajectory is difficult to demonstrate in the real world. Indeed, while some modelling studies provided strong support for tracing, their most supportive results were based on optimistic assumptions. Observational studies did also provide some support for contact tracing, but the certainty of this body of evidence was rated as very low. Policy-makers should keep these limitations in mind before enacting policies based on optimistic modelling studies, while researchers may wish to examine these and other potential confounders, mediators, and effect modifiers in future studies.

## Conclusions

Observational studies (*n* = 14) provided consistent, very-low certainty evidence that contact tracing (alone or in combination with other interventions) was associated with better control of COVID-19. Modelling studies (*n* = 18) provided consistent, high-certainty evidence that under assumptions of prompt and thorough tracing with effective quarantines, contact tracing could stop the spread of COVID-19. A cautious interpretation suggests that to stop the spread of COVID-19 with contact tracing, public health practitioners have 2–3 days from the time a new case develops symptoms to isolate the case and quarantine at least 80% of its contacts, and that once isolated, cases and contacts should infect zero new cases. Unfortunately, under assumptions of slower, less efficient tracing, contact tracing may slow, but not stop COVID-19. In those cases, given the limitations of this body of literature, it is unclear whether the benefits of tracing outweigh its costs, and practitioners may consider scaling down efforts as the US CDC advise [5], and turning instead to other more promising evidence-based, cost-effective interventions [2]. Future research may improve our understanding of contact tracing effectiveness by assessing emerging empirical evidence from ongoing efforts, best practices and policy responses, and differences in outcomes across jurisdictions with more or less efficient tracing.

## Funding

None.

## Authors' contributions

CEJ and ASB designed the study. CEJ and ASB searched the literature. CEJ, ASB, PC, and US analysed the literature. All authors interpreted the findings. CEJ and ASB wrote the first draft. All authors revised drafts and approved the final manuscript.

## Transparency declaration

The corresponding author (PC) affirms that this manuscript is an honest, accurate, and transparent account of the study being reported; that no important aspects of the study have been omitted; and that any discrepancies from the study as planned have been explained.

## Declaration of Competing Interest

The authors declare the following financial interests/personal relationships which may be considered as potential competing interests:

*CEJ has contractual agreements with the Centre intégré universitaire de santé et de services sociaux du Centre-Sud-de-l'Île-de-Montréal and is founder of the COVID-19 Science Updates.*

## Appendix A Supplementary data

Supplementary material 1 Image 1

Supplementary material 2 Image 2
