# Supplementary material for: Effective contact tracing for COVID-19: A systematic review
Source: Glob Epidemiol. 2023 Mar 9;5:100103. doi: 10.1016/j.gloepi.2023.100103 (PMC9997056; doi:10.1016/j.gloepi.2023.100103)
Supplement: Supplementary file 1 — Supplementary material 1 [file mmc1.docx]

**SUPPLEMENT—RESULT OF THE SEARCH**

| **Step** | **Searches** | **Results** |
| --- | --- | --- |
| 1 | COVID-19.mp. [mp=ti, ot, ab, nm, hw, kw, fx, kf, ox, px, rx, ui, an, sy, tx, sh, ct, tn, dm, mf, dv, dq, bt, id, cc] | 56118 |
| 2 | SARS-CoV-2.mp. [mp=ti, ot, ab, nm, hw, kw, fx, kf, ox, px, rx, ui, an, sy, tx, sh, ct, tn, dm, mf, dv, dq, bt, id, cc] | 17748 |
| 3 | 2019-NCoV.mp. [mp=ti, ot, ab, nm, hw, kw, fx, kf, ox, px, rx, ui, an, sy, tx, sh, ct, tn, dm, mf, dv, dq, bt, id, cc] | 1953 |
| 4 | coronavirus disease 2019.mp. [mp=ti, ot, ab, nm, hw, kw, fx, kf, ox, px, rx, ui, an, sy, tx, sh, ct, tn, dm, mf, dv, dq, bt, id, cc] | 29369 |
| 5 | severe acute respiratory syndrome coronavirus 2.mp. [mp=ti, ot, ab, nm, hw, kw, fx, kf, ox, px, rx, ui, an, sy, tx, sh, ct, tn, dm, mf, dv, dq, bt, id, cc] | 18947 |
| 6 | 2019 novel coronavirus.mp. [mp=ti, ot, ab, nm, hw, kw, fx, kf, ox, px, rx, ui, an, sy, tx, sh, ct, tn, dm, mf, dv, dq, bt, id, cc] | 1849 |
| 7 | 1 or 2 or 3 or 4 or 5 or 6 | 61984 |
| 8 | contact tracing.mp. [mp=ti, ot, ab, nm, hw, kw, fx, kf, ox, px, rx, ui, an, sy, tx, sh, ct, tn, dm, mf, dv, dq, bt, id, cc] | 9591 |
| 9 | contact-tracing.mp. [mp=ti, ot, ab, nm, hw, kw, fx, kf, ox, px, rx, ui, an, sy, tx, sh, ct, tn, dm, mf, dv, dq, bt, id, cc] | 9591 |
| 10 | tracing contact*.mp. [mp=ti, ot, ab, nm, hw, kw, fx, kf, ox, px, rx, ui, an, sy, tx, sh, ct, tn, dm, mf, dv, dq, bt, id, cc] | 79 |
| 11 | contact follow-up.mp. [mp=ti, ot, ab, nm, hw, kw, fx, kf, ox, px, rx, ui, an, sy, tx, sh, ct, tn, dm, mf, dv, dq, bt, id, cc] | 177 |
| 12 | case detection*.mp. [mp=ti, ot, ab, nm, hw, kw, fx, kf, ox, px, rx, ui, an, sy, tx, sh, ct, tn, dm, mf, dv, dq, bt, id, cc] | 8391 |
| 13 | contact investigation*.mp. [mp=ti, ot, ab, nm, hw, kw, fx, kf, ox, px, rx, ui, an, sy, tx, sh, ct, tn, dm, mf, dv, dq, bt, id, cc] | 2259 |
| 14 | epidemic investigation*.mp. [mp=ti, ot, ab, nm, hw, kw, fx, kf, ox, px, rx, ui, an, sy, tx, sh, ct, tn, dm, mf, dv, dq, bt, id, cc] | 267 |
| 15 | 8 or 9 or 10 or 11 or 12 or 13 or 14 | 19697 |
| 16 | 7 and 15 | 544 |
| 17 | remove duplicates from 16 | 343 |
