# Supplementary material for: Effective contact tracing for COVID-19: A systematic review
Source: Glob Epidemiol. 2023 Mar 9;5:100103. doi: 10.1016/j.gloepi.2023.100103 (PMC9997056; doi:10.1016/j.gloepi.2023.100103)
Supplement: Supplementary file 2 — Supplementary material 2 [file mmc2.pdf]

SUPPLEMENT

eFigure—PRISMA Flow Diagram

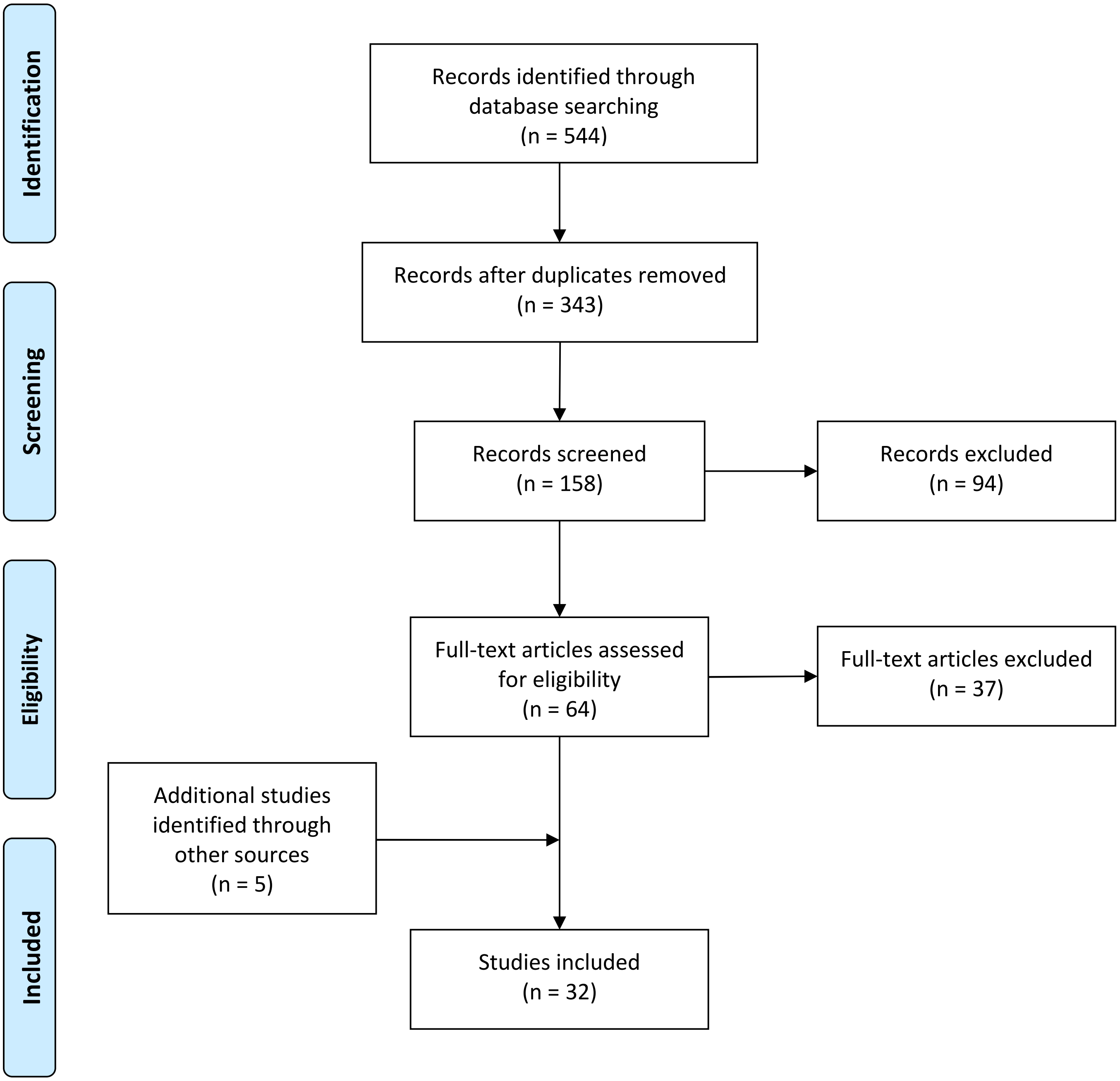

From: Moher D, Liberati A, Tetzlaff J, Altman DG, The PRISMA Group (2009). *P*referred *R*eporting *I*tems for *S*ystematic Reviews and *M*eta-*A*nalyses: The PRISMA Statement. PLoS Med 6(7): e1000097. doi:10.1371/journal.pmed1000097

For more information, visit www.prisma-statement.org.
